# (*E*)-9-(4-Chloro­styr­yl)-3,4,5,6,7,9-hexa­hydro-2*H*-xanthene-1,8-dione

**DOI:** 10.1107/S1600536812002139

**Published:** 2012-01-21

**Authors:** Jae Kyun Lee, Ae Nim Pae, Yong Seo Cho, Joo Hwan Cha

**Affiliations:** a Center for Neuro-Medicine, Korea Institute of Science & Technology, Hwarangro 14-gil, Seongbuk-gu, Seoul 136-791, Republic of Korea; b Advanced Analysis Center, Korea Institute of Science & Technology, Hwarangro 14-gil, Seongbuk-gu, Seoul 136-791, Republic of Korea

## Abstract

In the title compound, C_21_H_19_ClO_3_, the two cyclo­hexenone rings adopt half-chair conformations, whereas the pyran ring adopts a boat conformation. The 4-chloro­phenyl ring is almost perpendicular to the plane through the four C atoms of the pyran ring [dihedral angle = 87.97 (6)°]. In the crystal, weak C—H⋯O hydrogen bonds link the mol­ecules into a chain parallel to the *a*-axis.

## Related literature

For the biological activity of xanthenes and their derivatives, see: Lee *et al.* (2011 ▶). For related structures of xanthenes, see: Asad *et al.* (2012 ▶); Fun *et al.* (2011 ▶); Mehdi *et al.* (2011 ▶).
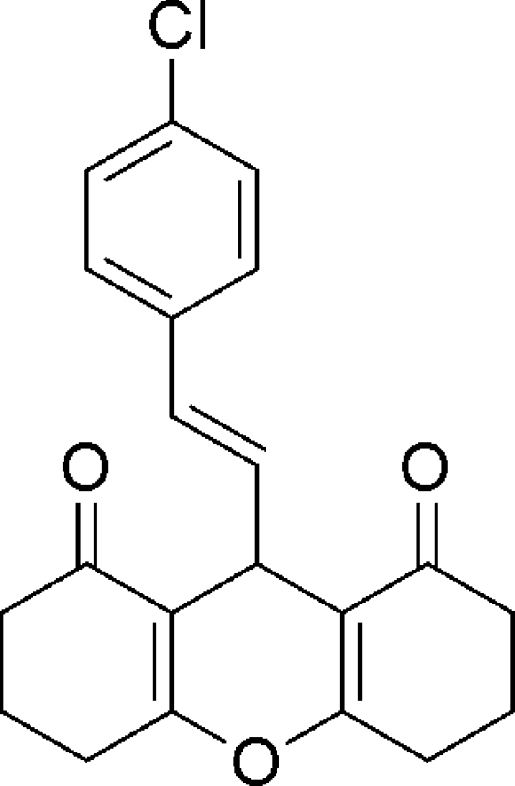


## Experimental

### 

#### Crystal data

C_21_H_19_ClO_3_
*M* *_r_* = 354.83
Monoclinic, 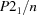

*a* = 5.6262 (7) Å
*b* = 16.273 (2) Å
*c* = 18.570 (3) Å
β = 90.125 (4)°
*V* = 1700.2 (4) Å^3^
*Z* = 4
Mo *K*α radiation
μ = 0.24 mm^−1^
*T* = 296 K
0.30 × 0.02 × 0.02 mm

#### Data collection

Rigaku R-AXIS RAPID diffractometer
Absorption correction: multi-scan (*ABSCOR*; Rigaku, 1995 ▶) *T*_min_ = 0.490, *T*_max_ = 0.995
13165 measured reflections
3066 independent reflections
1304 reflections with *F* ^2^ > 2σ(*F* ^2^)
*R*_int_ = 0.130

#### Refinement

*R*[*F* ^2^ > 2σ(*F* ^2^)] = 0.059
*wR*(*F* ^2^) = 0.157
*S* = 0.95
3066 reflections
234 parameters
H atoms treated by a mixture of independent and constrained refinement
Δρ_max_ = 0.30 e Å^−3^
Δρ_min_ = −0.24 e Å^−3^

### 

Data collection: *RAPID-AUTO* (Rigaku, 2006 ▶); cell refinement: *RAPID-AUTO*; data reduction: *RAPID-AUTO*; program(s) used to solve structure: *IL MILIONE* (Burla *et al.*, 2007 ▶); program(s) used to refine structure: *SHELXL97* (Sheldrick, 2008 ▶); molecular graphics: *CrystalStructure* (Rigaku, 2010 ▶) and *DIAMOND* (Brandenburg, 2006 ▶); software used to prepare material for publication: *CrystalStructure*.

## Supplementary Material

Crystal structure: contains datablock(s) global, I. DOI: 10.1107/S1600536812002139/kp2380sup1.cif

Structure factors: contains datablock(s) I. DOI: 10.1107/S1600536812002139/kp2380Isup2.hkl

Supplementary material file. DOI: 10.1107/S1600536812002139/kp2380Isup3.cml

Additional supplementary materials:  crystallographic information; 3D view; checkCIF report

## Figures and Tables

**Table 1:** Hydrogen-bond geometry (Å, °)

*D*—H⋯*A*	*D*—H	H⋯*A*	*D*⋯*A*	*D*—H⋯*A*
C18—H18*A*⋯O3^i^	0.97	2.50	3.316 (6)	142

Symmetry code: (i) 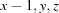.

**Fig. 1.**
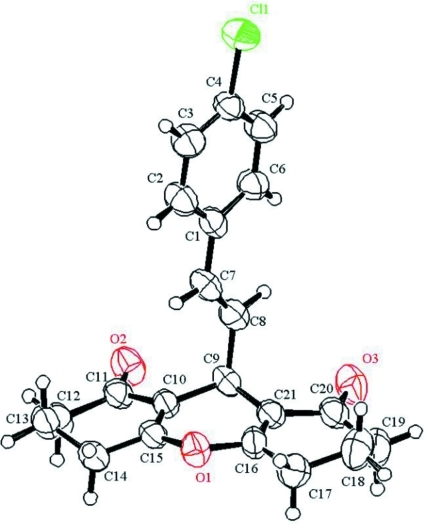
The molecular structure of (I) showing the atomic numbering and 50% probability displacement ellipsoid.

**Fig. 2.**
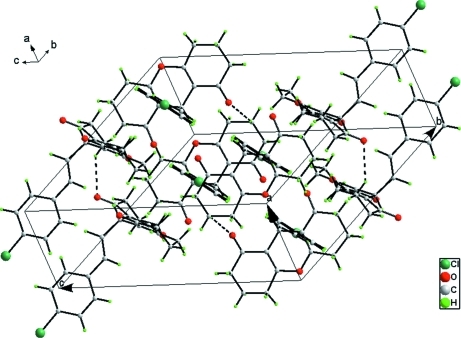
The crystal packing of the title compound with intermolecular contacts (dashed lines). The H atoms not involved in the intermolecular interactions have been omitted for clarity.

## supplementary crystallographic information

### Comment

As part of our ongoing study of the substituent effect on the solide state structures of xanthene derivatives (Lee *et al.*, 2011) the crystal structure of the title compound (I) (Fig. 1)is presented.

The bond lengths and angles are normal and correspond to those observed in related structures (Asad *et al.*, 2012; Fun *et al.*, 2011; Mehdi *et al.*, 2011). In the title compound, the dihedral angle between the 4-chlorophenyl and xanthenedione is 87.97 (6)°. Two cyclohexenone rings display a half-chair conformation whereas the pyran ring adopts a boat conformation. In the crystal packing (Fig. 2), intermolecular C18—H18A···O3 hydrogen bond (Table 1) link molecules into a chain.

### Experimental

To solution of (*E*))-2,2'-(3-(4-chlorophenyl)prop-2-ene-1,1-diyl)bis (3-hydroxycyclohex-2-enone) (1.25 mmol) methanol (12.5 ml) and catalytic amounts of sulfuric acid (0.2 ml) in under nitrogen atmosphere were added. After stirring for 3 h, the solvent was evaporated and the remaining residue was dissolved in ethyl acetate. The mixture was neutralized with saturated sodium bicarbonate and the solution was extracted with ethyl acetate. The resulting solid was purified by recrystallization from ethanol and methylene chloride to afford white needle crystals suitable for X-ray analysis.

### Refinement

All hydrogen atoms were positioned geometrically and refined using a riding model with C—H = 0.93–1.06 Å and *U*iso(H) = 1.2 or 1.5*U*eq(C).

### Crystal data

**Table:**

C_21_H_19_ClO_3_	*F*(000) = 744.00
*M**_r_* = 354.83	*D*_x_ = 1.386 Mg m^−^^3^
Monoclinic, *P*2_1_/*n*	Melting point: 474 K
Hall symbol: -P 2yn	Mo *K*α radiation, λ = 0.71075 Å
*a* = 5.6262 (7) Å	Cell parameters from 6227 reflections
*b* = 16.273 (2) Å	θ = 3.3–25.3°
*c* = 18.570 (3) Å	µ = 0.24 mm^−^^1^
β = 90.125 (4)°	*T* = 296 K
*V* = 1700.2 (4) Å^3^	Needle, yellow
*Z* = 4	0.30 × 0.02 × 0.02 mm

### Data collection

**Table:**

Rigaku R-AXIS RAPID diffractometer	1304 reflections with *F*^2^ > 2σ(*F*^2^)
Detector resolution: 10.000 pixels mm^-1^	*R*_int_ = 0.130
ω scans	θ_max_ = 25.3°
Absorption correction: multi-scan (*ABSCOR*; Rigaku, 1995)	*h* = −6→6
*T*_min_ = 0.490, *T*_max_ = 0.995	*k* = −19→19
13165 measured reflections	*l* = −22→22
3066 independent reflections	

### Refinement

**Table:**

Refinement on *F*^2^	Secondary atom site location: difference Fourier map
*R*[*F*^2^ > 2σ(*F*^2^)] = 0.059	Hydrogen site location: inferred from neighbouring sites
*wR*(*F*^2^) = 0.157	H atoms treated by a mixture of independent and constrained refinement
*S* = 0.95	*w* = 1/[σ^2^(*F*_o_^2^) + (0.0638*P*)^2^] where *P* = (*F*_o_^2^ + 2*F*_c_^2^)/3
3066 reflections	(Δ/σ)_max_ < 0.001
234 parameters	Δρ_max_ = 0.30 e Å^−^^3^
0 restraints	Δρ_min_ = −0.24 e Å^−^^3^
Primary atom site location: structure-invariant direct methods	

### Special details

**Table:**

Geometry. All e.s.d.'s (except the e.s.d. in the dihedral angle between two l.s. planes) are estimated using the full covariance matrix. The cell e.s.d.'s are taken into account individually in the estimation of e.s.d.'s in distances, angles and torsion angles; correlations between e.s.d.'s in cell parameters are only used when they are defined by crystal symmetry. An approximate (isotropic) treatment of cell e.s.d.'s is used for estimating e.s.d.'s involving l.s. planes.
Refinement. Refinement was performed using all reflections. The weighted *R*-factor (*wR*) and goodness of fit (*S*) are based on *F*^2^. *R*-factor (gt) are based on *F*. The threshold expression of *F*^2^ > 2.0 σ(*F*^2^) is used only for calculating *R*-factor (gt).

### Fractional atomic coordinates and isotropic or equivalent isotropic displacement parameters (Å^2^)

**Table:**

	*x*	*y*	*z*	*U*_iso_*/*U*_eq_	
Cl1	0.2944 (3)	0.68648 (7)	0.50318 (7)	0.0742 (5)	
O1	0.6470 (5)	0.24438 (17)	0.19262 (15)	0.0546 (8)	
O2	1.2810 (6)	0.43358 (19)	0.17692 (16)	0.0658 (9)	
O3	1.1501 (6)	0.23843 (19)	0.39585 (17)	0.0703 (10)	
C1	0.6501 (8)	0.5040 (3)	0.3556 (3)	0.0485 (11)	
C2	0.4387 (8)	0.5424 (3)	0.3366 (3)	0.0590 (12)	
C3	0.3346 (8)	0.6004 (3)	0.3809 (3)	0.0597 (12)	
C4	0.4386 (8)	0.6189 (3)	0.4455 (3)	0.0517 (11)	
C5	0.6509 (8)	0.5839 (3)	0.4652 (3)	0.0585 (12)	
C6	0.7543 (8)	0.5266 (3)	0.4206 (3)	0.0556 (12)	
C7	0.7497 (8)	0.4404 (3)	0.3081 (3)	0.0523 (12)	
C8	0.9399 (9)	0.3960 (3)	0.3186 (3)	0.0514 (11)	
C9	1.0229 (7)	0.3270 (3)	0.2694 (2)	0.0479 (11)	
C10	0.9648 (7)	0.3411 (3)	0.1915 (2)	0.0450 (11)	
C11	1.1125 (8)	0.3975 (3)	0.1491 (3)	0.0501 (11)	
C12	1.0565 (8)	0.4065 (3)	0.0708 (2)	0.0566 (12)	
C13	0.7925 (8)	0.3968 (3)	0.0548 (3)	0.0596 (13)	
C14	0.7031 (7)	0.3152 (3)	0.0832 (2)	0.0517 (11)	
C15	0.7850 (7)	0.3023 (3)	0.1583 (3)	0.0457 (11)	
C16	0.7265 (8)	0.2140 (3)	0.2576 (3)	0.0515 (12)	
C17	0.5817 (8)	0.1420 (3)	0.2807 (3)	0.0604 (13)	
C18	0.6238 (9)	0.1242 (3)	0.3605 (3)	0.0788 (16)	
C19	0.8789 (9)	0.1274 (3)	0.3801 (3)	0.0731 (15)	
C20	0.9904 (8)	0.2077 (3)	0.3599 (3)	0.0552 (12)	
C21	0.9076 (7)	0.2473 (3)	0.2938 (3)	0.0475 (11)	
H2	0.3657	0.5289	0.2932	0.0707*	
H3	0.1950	0.6266	0.3669	0.0716*	
H5	0.7242	0.5987	0.5082	0.0702*	
H6	0.8972	0.5024	0.4343	0.0667*	
H9	1.1956	0.3215	0.2743	0.0575*	
H12A	1.1445	0.3655	0.0439	0.0679*	
H12B	1.1083	0.4602	0.0546	0.0679*	
H13A	0.7049	0.4413	0.0773	0.0715*	
H13B	0.7660	0.3997	0.0033	0.0715*	
H14A	0.5308	0.3143	0.0816	0.0620*	
H14B	0.7614	0.2710	0.0530	0.0620*	
H17A	0.6251	0.0942	0.2523	0.0725*	
H17B	0.4146	0.1533	0.2725	0.0725*	
H18A	0.5375	0.1640	0.3891	0.0946*	
H18B	0.5617	0.0701	0.3719	0.0946*	
H19A	0.9622	0.0831	0.3560	0.0877*	
H19B	0.8954	0.1191	0.4317	0.0877*	
H8	1.046 (9)	0.408 (3)	0.364 (3)	0.12 (2)*	
H7	0.655 (7)	0.435 (3)	0.266 (3)	0.061 (13)*	

### Atomic displacement parameters (Å^2^)

**Table:**

	*U*^11^	*U*^22^	*U*^33^	*U*^12^	*U*^13^	*U*^23^
Cl1	0.0885 (10)	0.0694 (8)	0.0646 (9)	0.0114 (7)	0.0153 (7)	−0.0112 (7)
O1	0.0528 (17)	0.0641 (18)	0.047 (2)	−0.0088 (15)	−0.0037 (15)	0.0049 (16)
O2	0.062 (2)	0.084 (3)	0.052 (2)	−0.0199 (17)	−0.0020 (16)	0.0004 (17)
O3	0.069 (3)	0.089 (3)	0.053 (3)	0.0059 (18)	−0.0130 (17)	0.0064 (18)
C1	0.055 (3)	0.056 (3)	0.035 (3)	−0.009 (3)	0.005 (2)	−0.000 (2)
C2	0.053 (3)	0.076 (3)	0.048 (3)	0.007 (3)	−0.006 (3)	−0.008 (3)
C3	0.052 (3)	0.069 (3)	0.057 (4)	0.009 (3)	0.001 (3)	−0.001 (3)
C4	0.062 (3)	0.051 (3)	0.042 (3)	−0.004 (3)	0.012 (3)	−0.004 (3)
C5	0.063 (3)	0.065 (3)	0.048 (3)	−0.002 (3)	−0.008 (3)	−0.008 (3)
C6	0.057 (3)	0.060 (3)	0.050 (4)	0.011 (3)	−0.001 (3)	−0.003 (3)
C7	0.050 (3)	0.069 (3)	0.038 (3)	−0.007 (3)	−0.005 (3)	−0.011 (3)
C8	0.054 (3)	0.057 (3)	0.043 (3)	−0.005 (3)	−0.001 (3)	−0.005 (3)
C9	0.048 (3)	0.062 (3)	0.033 (3)	0.002 (2)	−0.0016 (19)	−0.005 (3)
C10	0.051 (3)	0.050 (3)	0.034 (3)	0.008 (2)	−0.000 (2)	0.000 (2)
C11	0.053 (3)	0.057 (3)	0.040 (3)	0.007 (3)	0.007 (3)	−0.002 (3)
C12	0.066 (3)	0.066 (3)	0.038 (3)	−0.000 (3)	0.002 (3)	0.004 (3)
C13	0.070 (4)	0.071 (3)	0.038 (3)	0.005 (3)	−0.006 (3)	0.003 (3)
C14	0.052 (3)	0.062 (3)	0.041 (3)	0.003 (3)	−0.001 (2)	−0.006 (3)
C15	0.048 (3)	0.053 (3)	0.036 (3)	0.004 (3)	0.003 (2)	−0.004 (2)
C16	0.061 (3)	0.056 (3)	0.038 (3)	0.011 (3)	0.003 (3)	0.009 (3)
C17	0.063 (3)	0.059 (3)	0.060 (4)	−0.007 (3)	0.008 (3)	0.002 (3)
C18	0.084 (4)	0.076 (4)	0.076 (4)	−0.001 (3)	0.010 (3)	0.025 (3)
C19	0.083 (4)	0.073 (4)	0.062 (4)	0.011 (3)	0.005 (3)	0.020 (3)
C20	0.062 (3)	0.063 (3)	0.041 (3)	0.014 (3)	0.007 (3)	−0.001 (3)
C21	0.052 (3)	0.053 (3)	0.037 (3)	0.010 (3)	0.001 (2)	−0.002 (3)

### Geometric parameters (Å, °)

**Table:**

Cl1—C4	1.737 (5)	C17—C18	1.529 (7)
O1—C15	1.377 (5)	C18—C19	1.481 (7)
O1—C16	1.379 (6)	C19—C20	1.499 (7)
O2—C11	1.228 (6)	C20—C21	1.461 (6)
O3—C20	1.225 (6)	C2—H2	0.930
C1—C2	1.388 (6)	C3—H3	0.930
C1—C6	1.391 (6)	C5—H5	0.930
C1—C7	1.472 (6)	C6—H6	0.930
C2—C3	1.383 (7)	C7—H7	0.96 (4)
C3—C4	1.368 (7)	C8—H8	1.06 (6)
C4—C5	1.372 (7)	C9—H9	0.980
C5—C6	1.376 (6)	C12—H12A	0.970
C7—C8	1.306 (7)	C12—H12B	0.970
C8—C9	1.520 (6)	C13—H13A	0.970
C9—C10	1.501 (6)	C13—H13B	0.970
C9—C21	1.520 (6)	C14—H14A	0.970
C10—C11	1.468 (6)	C14—H14B	0.970
C10—C15	1.341 (6)	C17—H17A	0.970
C11—C12	1.495 (6)	C17—H17B	0.970
C12—C13	1.522 (6)	C18—H18A	0.970
C13—C14	1.515 (6)	C18—H18B	0.970
C14—C15	1.483 (6)	C19—H19A	0.970
C16—C17	1.491 (6)	C19—H19B	0.970
C16—C21	1.335 (6)		
			
O1···C9	2.881 (5)	C11···H18B^vi^	3.0007
O2···C8	3.317 (6)	C12···H12B^xi^	3.3132
O2···C9	2.842 (5)	C12···H13B^xi^	3.5843
O2···C15	3.531 (5)	C12···H14A^ii^	3.0675
O3···C8	3.165 (6)	C12···H18A^xii^	3.5651
O3···C9	2.845 (5)	C12···H18B^vi^	2.9442
O3···C16	3.521 (6)	C12···H19B^xii^	3.2416
O3···C18	3.556 (6)	C13···H12B^xi^	3.1400
C1···C4	2.777 (6)	C13···H18A^xii^	3.5169
C2···C5	2.752 (7)	C13···H18B^vi^	3.2365
C3···C6	2.749 (6)	C13···H19B^v^	3.2044
C6···C8	3.034 (6)	C14···H12A^iv^	3.3275
C7···C10	2.962 (6)	C14···H19B^v^	3.4699
C7···C15	3.581 (7)	C16···H9^iv^	3.4764
C7···C21	3.275 (6)	C17···H2^viii^	3.4042
C8···C11	3.296 (7)	C17···H3^viii^	3.1582
C8···C15	3.455 (6)	C18···H12A^xiii^	3.4111
C8···C16	3.389 (6)	C18···H12B^xiv^	3.3639
C8···C20	3.170 (6)	C18···H13A^xiv^	3.3343
C10···C13	2.862 (6)	C18···H13B^xiii^	3.3548
C10···C16	2.756 (6)	C19···H12A^xiii^	3.3187
C11···C14	2.930 (6)	C19···H13A^xiv^	3.1656
C12···C15	2.804 (6)	C19···H13B^iii^	3.1845
C15···C21	2.757 (6)	C20···H13B^iii^	3.5396
C16···C19	2.809 (7)	C20···H17B^ii^	3.0220
C17···C20	2.929 (7)	C20···H18A^ii^	3.2042
C18···C21	2.847 (7)	C21···H17B^ii^	3.2623
Cl1···O3^i^	3.359 (4)	H2···O2^iv^	2.7005
Cl1···C20^i^	3.467 (5)	H2···C8^iv^	3.2630
O2···C2^ii^	3.565 (6)	H2···C17^vii^	3.4042
O2···C7^ii^	3.588 (6)	H2···H9^iv^	3.5252
O2···C14^ii^	3.521 (5)	H2···H17A^vii^	3.0753
O2···C15^ii^	3.568 (5)	H2···H17A^vi^	3.1724
O3···Cl1^i^	3.359 (4)	H2···H17B^vii^	2.8393
O3···C14^iii^	3.599 (5)	H2···H19A^vi^	3.0672
O3···C18^ii^	3.317 (6)	H2···H8^iv^	2.9741
C2···O2^iv^	3.565 (6)	H3···O1^vii^	2.9310
C3···C6^iv^	3.557 (6)	H3···C6^iv^	3.1309
C5···C5^i^	3.466 (6)	H3···C17^vii^	3.1582
C5···C6^i^	3.598 (6)	H3···H6^iv^	2.9096
C6···C3^ii^	3.557 (6)	H3···H14A^vii^	3.4449
C6···C5^i^	3.598 (6)	H3···H17A^vii^	2.8986
C7···O2^iv^	3.588 (6)	H3···H17B^vii^	2.6940
C14···O2^iv^	3.521 (5)	H5···Cl1^ii^	3.5138
C14···O3^v^	3.599 (5)	H5···O3^x^	3.2704
C15···O2^iv^	3.568 (5)	H5···H6^x^	2.8933
C18···O3^iv^	3.317 (6)	H5···H14B^vi^	3.0276
C20···Cl1^i^	3.467 (5)	H5···H8^x^	2.6944
Cl1···H3	2.7686	H6···Cl1^i^	3.4592
Cl1···H5	2.8101	H6···C3^ii^	3.0969
O1···H14A	2.4421	H6···C4^ii^	3.5932
O1···H14B	2.7084	H6···C4^i^	3.5306
O1···H17A	2.6866	H6···C5^x^	3.4489
O1···H17B	2.4731	H6···C6^x^	3.3615
O1···H7	3.38 (4)	H6···H3^ii^	2.9096
O2···H9	2.6141	H6···H5^x^	2.8933
O2···H12A	2.8131	H6···H6^x^	2.6985
O2···H12B	2.5062	H9···O1^ii^	3.2165
O3···H9	2.6434	H9···C16^ii^	3.4764
O3···H19A	2.8377	H9···H2^ii^	3.5252
O3···H19B	2.5037	H9···H17B^ii^	3.0027
O3···H8	2.88 (5)	H9···H7^ii^	3.1772
C1···H3	3.2526	H12A···Cl1^xiv^	3.0610
C1···H5	3.2525	H12A···C14^ii^	3.3275
C1···H8	2.72 (5)	H12A···C18^xii^	3.4111
C2···H6	3.2173	H12A···C19^xii^	3.3187
C2···H7	2.51 (4)	H12A···H13A^ii^	3.4409
C3···H5	3.2211	H12A···H14A^ii^	2.4297
C4···H2	3.2107	H12A···H18A^xii^	2.9741
C4···H6	3.2087	H12A···H18B^xii^	3.3927
C5···H3	3.2214	H12A···H19B^xii^	2.5322
C6···H2	3.2183	H12B···C12^xi^	3.3132
C6···H8	2.74 (5)	H12B···C13^xi^	3.1400
C6···H7	3.29 (4)	H12B···C18^vi^	3.3639
C7···H2	2.6112	H12B···H12B^xi^	2.6951
C7···H6	2.6827	H12B···H13A^ii^	3.3961
C7···H9	3.2300	H12B···H13A^xi^	3.1116
C8···H6	2.7711	H12B···H13B^xi^	2.6186
C9···H7	2.71 (4)	H12B···H14A^ii^	3.3965
C10···H12A	2.9504	H12B···H18A^vi^	3.5734
C10···H12B	3.2984	H12B···H18B^vi^	2.4456
C10···H13A	3.0459	H12B···H18B^xii^	3.4387
C10···H14A	3.2078	H12B···H19A^ix^	3.5456
C10···H14B	3.0353	H12B···H19B^xii^	3.0834
C10···H8	3.42 (6)	H13A···O2^iv^	3.0247
C10···H7	2.70 (4)	H13A···C18^vi^	3.3343
C11···H9	2.6738	H13A···C19^vi^	3.1656
C11···H13A	2.7447	H13A···H12A^iv^	3.4409
C11···H13B	3.3340	H13A···H12B^iv^	3.3961
C11···H14B	3.3630	H13A···H12B^xi^	3.1116
C11···H7	3.42 (4)	H13A···H18B^vi^	2.6469
C12···H14A	3.3231	H13A···H19A^vi^	2.7840
C12···H14B	2.7793	H13A···H19B^vi^	2.9537
C14···H12A	2.7165	H13A···H19B^v^	3.3601
C14···H12B	3.3254	H13B···O3^v^	3.0736
C15···H9	3.1710	H13B···C12^xi^	3.5843
C15···H12A	3.1122	H13B···C18^xii^	3.3548
C15···H13A	2.7537	H13B···C19^v^	3.1845
C15···H13B	3.2884	H13B···C20^v^	3.5396
C15···H7	3.03 (4)	H13B···H12B^xi^	2.6186
C16···H9	3.1811	H13B···H18A^xii^	2.8134
C16···H18A	2.7863	H13B···H18B^xii^	2.9969
C16···H18B	3.2940	H13B···H19A^v^	3.2336
C16···H19A	3.1021	H13B···H19B^v^	2.4891
C17···H19A	2.7286	H14A···Cl1^viii^	3.1848
C17···H19B	3.3306	H14A···O2^iv^	2.9806
C19···H17A	2.8197	H14A···C11^iv^	2.9919
C19···H17B	3.3132	H14A···C12^iv^	3.0675
C20···H9	2.7008	H14A···H3^viii^	3.4449
C20···H17A	3.4068	H14A···H12A^iv^	2.4297
C20···H18A	2.7016	H14A···H12B^iv^	3.3965
C20···H18B	3.2989	H14A···H19B^v^	3.0826
C20···H8	3.28 (5)	H14B···Cl1^viii^	3.5700
C21···H17A	3.0540	H14B···Cl1^xiv^	3.0390
C21···H17B	3.1924	H14B···O3^v^	2.9866
C21···H18A	3.0528	H14B···C4^xiv^	2.9960
C21···H19A	2.9276	H14B···C5^xiv^	3.1038
C21···H19B	3.3026	H14B···H5^xiv^	3.0276
C21···H8	3.03 (5)	H14B···H18A^xii^	3.5788
C21···H7	3.40 (4)	H14B···H19B^v^	3.5342
H2···H3	2.3074	H17A···C1^xiv^	2.7895
H2···H7	2.2949	H17A···C2^xiv^	3.0787
H5···H6	2.3001	H17A···C3^viii^	3.5763
H6···H8	2.1765	H17A···C6^xiv^	3.4635
H6···H7	3.5875	H17A···C7^xiv^	2.8328
H9···H8	2.3468	H17A···C8^xiv^	3.5036
H9···H7	3.5592	H17A···H2^viii^	3.0753
H12A···H13A	2.8331	H17A···H2^xiv^	3.1724
H12A···H13B	2.3250	H17A···H3^viii^	2.8986
H12A···H14B	2.6532	H17A···H7^xiv^	2.8962
H12B···H13A	2.3297	H17B···O3^iv^	3.0658
H12B···H13B	2.3630	H17B···C2^viii^	3.3599
H13A···H14A	2.2882	H17B···C3^viii^	3.2863
H13A···H14B	2.8251	H17B···C9^iv^	3.5857
H13A···H7	3.5115	H17B···C20^iv^	3.0220
H13B···H14A	2.4098	H17B···C21^iv^	3.2623
H13B···H14B	2.2880	H17B···H2^viii^	2.8393
H17A···H18A	2.8272	H17B···H3^viii^	2.6940
H17A···H18B	2.2832	H17B···H9^iv^	3.0027
H17A···H19A	2.7061	H17B···H19A^iv^	3.1948
H17B···H18A	2.2790	H18A···Cl1^i^	3.2869
H17B···H18B	2.4325	H18A···O3^iv^	2.4963
H18A···H19A	2.7983	H18A···C12^xiii^	3.5651
H18A···H19B	2.2821	H18A···C13^xiii^	3.5169
H18B···H19A	2.2829	H18A···C20^iv^	3.2042
H18B···H19B	2.3207	H18A···H12A^xiii^	2.9741
H8···H7	2.89 (7)	H18A···H12B^xiv^	3.5734
Cl1···H5^iv^	3.5138	H18A···H13B^xiii^	2.8134
Cl1···H6^i^	3.4592	H18A···H14B^xiii^	3.5788
Cl1···H12A^vi^	3.0610	H18A···H19A^iv^	3.5469
Cl1···H14A^vii^	3.1848	H18B···O2^xiv^	3.0768
Cl1···H14B^vii^	3.5700	H18B···C11^xiv^	3.0007
Cl1···H14B^vi^	3.0390	H18B···C12^xiv^	2.9442
Cl1···H18A^i^	3.2869	H18B···C13^xiv^	3.2365
Cl1···H19B^i^	3.5522	H18B···H12A^xiii^	3.3927
Cl1···H8^i^	3.48 (6)	H18B···H12B^xiv^	2.4456
O1···H3^viii^	2.9310	H18B···H12B^xiii^	3.4387
O1···H9^iv^	3.2165	H18B···H13A^xiv^	2.6469
O2···H2^ii^	2.7005	H18B···H13B^xiii^	2.9969
O2···H13A^ii^	3.0247	H18B···H19A^iv^	3.3917
O2···H14A^ii^	2.9806	H19A···O2^xv^	2.8956
O2···H18B^vi^	3.0768	H19A···H2^xiv^	3.0672
O2···H19A^ix^	2.8956	H19A···H12B^xv^	3.5456
O2···H7^ii^	2.67 (4)	H19A···H13A^xiv^	2.7840
O3···H5^x^	3.2704	H19A···H13B^iii^	3.2336
O3···H13B^iii^	3.0736	H19A···H17B^ii^	3.1948
O3···H14B^iii^	2.9866	H19A···H18A^ii^	3.5469
O3···H17B^ii^	3.0658	H19A···H18B^ii^	3.3917
O3···H18A^ii^	2.4963	H19A···H7^xiv^	3.3714
C1···H17A^vi^	2.7895	H19B···Cl1^i^	3.5522
C2···H17A^vi^	3.0787	H19B···C12^xiii^	3.2416
C2···H17B^vii^	3.3599	H19B···C13^iii^	3.2044
C2···H8^iv^	3.15 (5)	H19B···C14^iii^	3.4699
C3···H6^iv^	3.0969	H19B···H12A^xiii^	2.5322
C3···H17A^vii^	3.5763	H19B···H12B^xiii^	3.0834
C3···H17B^vii^	3.2863	H19B···H13A^xiv^	2.9537
C3···H8^iv^	3.54 (5)	H19B···H13A^iii^	3.3601
C4···H6^iv^	3.5932	H19B···H13B^iii^	2.4891
C4···H6^i^	3.5306	H19B···H14A^iii^	3.0826
C4···H14B^vi^	2.9960	H19B···H14B^iii^	3.5342
C5···H6^x^	3.4489	H8···Cl1^i^	3.48 (6)
C5···H14B^vi^	3.1038	H8···C2^ii^	3.15 (5)
C5···H8^x^	3.59 (6)	H8···C3^ii^	3.54 (5)
C6···H3^ii^	3.1309	H8···C5^x^	3.59 (6)
C6···H6^x^	3.3615	H8···H2^ii^	2.9741
C6···H17A^vi^	3.4635	H8···H5^x^	2.6944
C7···H17A^vi^	2.8328	H7···O2^iv^	2.67 (4)
C8···H2^ii^	3.2630	H7···H9^iv^	3.1772
C8···H17A^vi^	3.5036	H7···H17A^vi^	2.8962
C9···H17B^ii^	3.5857	H7···H19A^vi^	3.3714
C11···H14A^ii^	2.9919		
			
C15—O1—C16	117.9 (3)	C4—C3—H3	120.295
C2—C1—C6	117.4 (4)	C4—C5—H5	120.343
C2—C1—C7	119.5 (4)	C6—C5—H5	120.335
C6—C1—C7	123.1 (4)	C1—C6—H6	119.226
C1—C2—C3	121.3 (4)	C5—C6—H6	119.214
C2—C3—C4	119.4 (4)	C1—C7—H7	110 (3)
Cl1—C4—C3	118.7 (4)	C8—C7—H7	122 (3)
Cl1—C4—C5	120.4 (4)	C7—C8—H8	118 (3)
C3—C4—C5	120.9 (4)	C9—C8—H8	117 (3)
C4—C5—C6	119.3 (4)	C8—C9—H9	108.517
C1—C6—C5	121.6 (4)	C10—C9—H9	108.517
C1—C7—C8	127.8 (5)	C21—C9—H9	108.517
C7—C8—C9	124.8 (4)	C11—C12—H12A	109.090
C8—C9—C10	113.6 (4)	C11—C12—H12B	109.095
C8—C9—C21	108.6 (4)	C13—C12—H12A	109.096
C10—C9—C21	109.0 (4)	C13—C12—H12B	109.096
C9—C10—C11	119.3 (4)	H12A—C12—H12B	107.839
C9—C10—C15	122.2 (4)	C12—C13—H13A	109.587
C11—C10—C15	118.4 (4)	C12—C13—H13B	109.587
O2—C11—C10	120.7 (4)	C14—C13—H13A	109.580
O2—C11—C12	121.5 (4)	C14—C13—H13B	109.577
C10—C11—C12	117.7 (4)	H13A—C13—H13B	108.127
C11—C12—C13	112.5 (4)	C13—C14—H14A	109.571
C12—C13—C14	110.3 (4)	C13—C14—H14B	109.570
C13—C14—C15	110.4 (4)	C15—C14—H14A	109.565
O1—C15—C10	122.4 (4)	C15—C14—H14B	109.565
O1—C15—C14	111.0 (4)	H14A—C14—H14B	108.112
C10—C15—C14	126.7 (4)	C16—C17—H17A	109.647
O1—C16—C17	110.9 (4)	C16—C17—H17B	109.641
O1—C16—C21	122.8 (4)	C18—C17—H17A	109.632
C17—C16—C21	126.3 (4)	C18—C17—H17B	109.638
C16—C17—C18	110.1 (4)	H17A—C17—H17B	108.161
C17—C18—C19	112.4 (5)	C17—C18—H18A	109.137
C18—C19—C20	112.0 (4)	C17—C18—H18B	109.133
O3—C20—C19	121.7 (4)	C19—C18—H18A	109.133
O3—C20—C21	120.6 (4)	C19—C18—H18B	109.127
C19—C20—C21	117.6 (4)	H18A—C18—H18B	107.854
C9—C21—C16	121.5 (4)	C18—C19—H19A	109.215
C9—C21—C20	119.5 (4)	C18—C19—H19B	109.211
C16—C21—C20	119.0 (4)	C20—C19—H19A	109.213
C1—C2—H2	119.337	C20—C19—H19B	109.201
C3—C2—H2	119.331	H19A—C19—H19B	107.906
C2—C3—H3	120.285		
			
C15—O1—C16—C17	−169.7 (3)	C9—C10—C11—C12	176.9 (3)
C15—O1—C16—C21	11.3 (6)	C9—C10—C15—O1	−2.6 (6)
C16—O1—C15—C10	−13.3 (5)	C9—C10—C15—C14	176.0 (4)
C16—O1—C15—C14	167.9 (3)	C11—C10—C15—O1	175.7 (4)
C2—C1—C6—C5	1.0 (6)	C11—C10—C15—C14	−5.7 (6)
C6—C1—C2—C3	−0.6 (6)	C15—C10—C11—O2	−179.0 (4)
C2—C1—C7—C8	−177.1 (4)	C15—C10—C11—C12	−1.4 (6)
C7—C1—C2—C3	177.7 (4)	O2—C11—C12—C13	−150.2 (4)
C6—C1—C7—C8	1.0 (7)	C10—C11—C12—C13	32.2 (5)
C7—C1—C6—C5	−177.2 (4)	C11—C12—C13—C14	−55.7 (5)
C1—C2—C3—C4	−1.6 (7)	C12—C13—C14—C15	47.7 (4)
C2—C3—C4—Cl1	−175.5 (4)	C13—C14—C15—O1	160.2 (3)
C2—C3—C4—C5	3.4 (7)	C13—C14—C15—C10	−18.6 (6)
Cl1—C4—C5—C6	175.9 (3)	O1—C16—C17—C18	−164.2 (3)
C3—C4—C5—C6	−3.0 (7)	O1—C16—C21—C9	6.5 (6)
C4—C5—C6—C1	0.7 (6)	O1—C16—C21—C20	−175.8 (3)
C1—C7—C8—C9	175.5 (4)	C17—C16—C21—C9	−172.5 (4)
C7—C8—C9—C10	32.5 (6)	C17—C16—C21—C20	5.3 (7)
C7—C8—C9—C21	−88.9 (5)	C21—C16—C17—C18	14.8 (6)
C8—C9—C10—C11	78.4 (5)	C16—C17—C18—C19	−44.7 (5)
C8—C9—C10—C15	−103.2 (4)	C17—C18—C19—C20	55.4 (5)
C8—C9—C21—C16	104.4 (4)	C18—C19—C20—O3	146.9 (5)
C8—C9—C21—C20	−73.3 (5)	C18—C19—C20—C21	−35.1 (6)
C10—C9—C21—C16	−19.8 (5)	O3—C20—C21—C9	0.6 (6)
C10—C9—C21—C20	162.5 (3)	O3—C20—C21—C16	−177.2 (4)
C21—C9—C10—C11	−160.4 (3)	C19—C20—C21—C9	−177.4 (4)
C21—C9—C10—C15	18.0 (5)	C19—C20—C21—C16	4.8 (6)
C9—C10—C11—O2	−0.7 (6)		

Symmetry codes: (i) −*x*+1, −*y*+1, −*z*+1; (ii) *x*+1, *y*, *z*; (iii) *x*+1/2, −*y*+1/2, *z*+1/2; (iv) *x*−1, *y*, *z*; (v) *x*−1/2, −*y*+1/2, *z*−1/2; (vi) −*x*+3/2, *y*+1/2, −*z*+1/2; (vii) −*x*+1/2, *y*+1/2, −*z*+1/2; (viii) −*x*+1/2, *y*−1/2, −*z*+1/2; (ix) −*x*+5/2, *y*+1/2, −*z*+1/2; (x) −*x*+2, −*y*+1, −*z*+1; (xi) −*x*+2, −*y*+1, −*z*; (xii) *x*+1/2, −*y*+1/2, *z*−1/2; (xiii) *x*−1/2, −*y*+1/2, *z*+1/2; (xiv) −*x*+3/2, *y*−1/2, −*z*+1/2; (xv) −*x*+5/2, *y*−1/2, −*z*+1/2.

### Hydrogen-bond geometry (Å, °)

**Table:**

*D*—H···*A*	*D*—H	H···*A*	*D*···*A*	*D*—H···*A*
C18—H18A···O3^iv^	0.97	2.50	3.316 (6)	142.

Symmetry codes: (iv) *x*−1, *y*, *z*.
